# Effect of Blueberry Pomace Addition on Quality Attributes of Buttermilk-Based Fermented Drinks during Cold Storage

**DOI:** 10.3390/foods13111770

**Published:** 2024-06-05

**Authors:** Biljana Trajkovska, Gjore Nakov, Sari Thachappully Prabhat, Prarabdh C. Badgujar

**Affiliations:** 1 Faculty of Biotechnical Sciences—Bitola, University “St. Kliment Ohridski”—Bitola, 7000 Bitola, North Macedonia; 2 College of Sliven, Technical University of Sofia, 8800 Sliven, Bulgaria; gnakov@tu-sofia.bg; 3 Department of Food Science and Technology, National Institute of Food Technology Entrepreneurship and Management, Kundli, Sonipat 131028, Haryana, India; sari.tp.tp@gmail.com (S.T.P.); prarabdh.badgujar@gmail.com (P.C.B.)

**Keywords:** buttermilk, blueberry pomace, physicochemical properties, antioxidant activity, microbiological analysis, sensory evaluation

## Abstract

The fruit and beverage industry faces challenges related to waste management and environmental pollution due to rapid industrial expansion. Fruit industry waste, such as blueberry pomace, holds the promise of enhancing gut health and providing valuable antioxidants. Concurrently, buttermilk, a prominent dairy product, offers nutritional and technological benefits but remains underutilized. This study aimed to evaluate the incorporation of blueberry pomace (0%, 2%, 4%, 6%, 8%, and 10%) into buttermilk at varying levels and assess its impact on the physicochemical, antioxidant, microbiological, and sensory characteristics of the buttermilk. Buttermilk samples were supplemented with different concentrations of blueberry pomace and subjected to analysis over a two-week storage period (4 ± 1 °C). The addition of blueberry pomace led to alterations in the pH, dry matter, water holding capacity, color parameters, total phenolic content, and antioxidant activity. Microbiological analysis revealed the absence of *Enterobacteriaceae*, yeast, or molds. Sensory evaluation indicated significant differences among samples, with the highest scores observed for the buttermilk supplemented with 2% and 4% blueberry pomace. Incorporating blueberry pomace improved the overall acceptability and sensory properties. This research highlights the potential of fruit industry by-products to enhance the functionality and health benefits of dairy products, which is a promising way to effectively utilize waste.

## 1. Introduction

The surge in global demand for dairy products has propelled the dairy sector’s growth, transitioning from traditional to mechanized production and scaling up to meet consumer needs. However, this rapid industrial expansion not only leads to increased production but also raises concerns about higher concentrations of pollutants in water and land, contributing to environmental pollution and potential health risks [1]. By-products from the dairy industry pose a significant environmental threat due to their high organic compound content [2]. Buttermilk (BM) stands out as a major by-product in the dairy industry, formed in the serum phase during butter production [3]. Despite its nutritional and technological merits, BM remains underutilized [4]. Its composition typically includes lactose, proteins (casein and serum proteins), lipids, ash, and polar lipids (phospholipids and sphingolipids) originating from the milk fat globule membrane (MFGM). Notably, the concentration of polar lipids in BM is about five times higher than in whole milk [5,6]. Various types of BM are produced, including cultured buttermilk, sweet cream buttermilk, sour cream buttermilk, and commercial buttermilk [7]. On the other hand, dairy products are frequently enriched with a variety of ingredients such as fruit [8] and press cake flour [9,10] to amplify their positive health-promoting effects.

The agro-food industry has witnessed a notable increase in the production of by-products in recent years, which offer the potential for added value due to their functional and/or bioactive properties, promoting the concept of a circular economy [11]. Blueberry pomace (BP), a by-product of the juice industry, comprises seeds, skins, and pulp residue, constituting 20–30% of the fruit [12]. With its retained phenolic compounds and dietary fiber content, BP holds promise for enhancing gut health, potentially influencing gut microbiota composition [13]. Fermentation of BP by the probiotic *Lactobacillus casei* has been shown to enhance antioxidant activity and regulate fecal microbiota, offering potential health benefits [14]. Moreover, fruit by-products such as blueberry pomace are rich in bioactive compounds such as polyphenols, anthocyanins, phenolic acids, flavanols, and tannins, making them valuable sources of antioxidants [15,16]. These compounds exhibit significant antimicrobial activity, offering potential as innovative natural food additives [17].

The objective of this research was to evaluate the impact of incorporating BP, a fruit by-product, into BM at various levels (2%, 4%, 6%, 8%, and 10%) and to examine its effects on the physicochemical, technological, and sensory characteristics of the product. This study aimed at valorizing fruit by-products by incorporating them into BM to create a functional dairy product.

## 2. Materials and Methods

### 2.1. Materials and Buttermilk Production

Approximately 20 L of BM underwent chemical composition analysis (MilkoScanTM FT3, Foss, Hilleroed, Denmark). Before producing the various types of fortified BM-based fermented drinks, the chemical composition of the BM utilized was analyzed. The composition was as follows: 0.94% fat, 3.43% protein, 4.81% lactose, 9.18% dry matter, and a pH of 6.53. Additionally, we conducted a microbiological screening of the BM prior to fortification. The results showed that yeast and molds were not detected, *Escherichia coli* was absent (0 CFU/mL), the *Enterobacteriaceae* count was less than 10 CFU/mL, and the total bacterial/plate count was less than 100 CFU/mL. These analyses ensured the quality and safety of the BM before using it to produce fortified fermented drinks.

Following this, BM underwent thermal treatment at 72 ± 1 °C (Weck Inc., Luray, VA, USA) for 10 min, as described by Szkolnicka et al. [18]. After pasteurization, the BM was cooled to 35 ± 1 °C at room temperature. Mesophilic starter cultures (Selection TM Danica, CHR Hansen, Hørsholm, Denmark) were then added according to the manufacturer’s guidelines (500 U/5000 L). The bacterial strains were *Lactococcus lactis* subsp. *cremoris*, *Lactococcus lactis* subsp. *lactis*, *Lactococcus lactis* subsp. *lactis biovar*, *Diacetylactis*, and *Leuconostoc*. These lactic acid bacteria are known for their ability to produce aroma and CO_2_.

BP, obtained as a by-product from juice processing, was dried in a convection oven at 45 ± 2 °C for 20 h, ground, and passed through a 1 mm sieve [16]. The BP composition was as follows: 0.5 g/100 g fats, 87 g/100 g carbohydrates, 5 g/100 g fibers, and 0.2 g/100 g proteins. Various quantities of BP (2 g, 4 g, 6 g, 8 g, and 10 g per 100 g of BM) were added to sterile containers and the inoculated BM was divided among them, mixed, and incubated (yogurt maker Y 140, Elecrem, UK) at a constant temperature of 35 ± 1 °C. Incubation continued until the pH of the control BM samples without BP reached 4.6 ± 0.1 (9 h), at which point incubation was halted for all samples. Throughout the fermentation, the pH of the milk was monitored using a pH meter (Testo SE & Co., KGaA, Lenzkirch, Germany). After incubation, the BM samples were refrigerated (4 ± 1 °C) for subsequent analysis. Physicochemical and microbiological properties were evaluated over a 2-week period, with measurements taken weekly (on days 1, 7, and 14) during refrigerated storage at 4 ± 1 °C. Sensorial properties were evaluated only on the first day after production.

**Figure 1:**
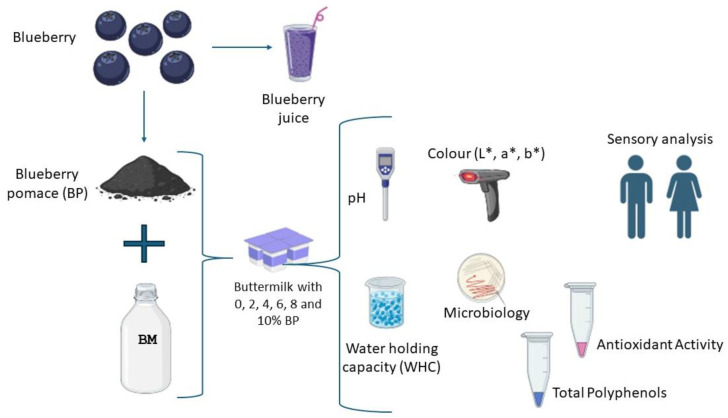
Experimental workflow for incorporating blueberry pomace into buttermilk and evaluating physicochemical, microbiological, and sensory properties.

All BM-based fermented drinks were prepared in triplicate batches. Control samples without BP were referred to as plain samples, while those with BP were considered fortified samples (Figure 1).

### 2.2. Methods

#### 2.2.1. pH Determination

The pH of the samples was measured at 20 °C using a pH meter (Testo SE & Co., KGaA, Lenzkirch, Germany) that was prior calibrated with pH 4.0 and 7.0 buffers.

#### 2.2.2. Dry Matter

Two grams (2 g) of BM-based fermented drinks were weighed in an aluminum dish and dried at 102 ± 1 °C until a constant weight was achieved using a moisture analyzer MJ33 (Mettler Toledo, Greifensee, Switzerland) [19].

#### 2.2.3. Water Holding Capacity and Color Determination

The water holding capacity (WHC) of the BM-based fermented samples was assessed using the centrifugation method, as per Grasso et al. [20]. The color of the BM-based fermented drink was evaluated using a colorimeter (Konica Minolta, Chroma Meter, CR400, Osaka, Japan), following the methodology outlined by Nakov et al. [9]. Parameters such as L* value (lightness), a* value (red-green intensity), and b* value (yellow-blue intensity) were measured for the BM samples. The total color change (ΔE) between the control sample and the BM-based fermented drink with different BP content was also determined: *L*_1_*, a*_1_, and *b*_1_ are color parameters for the control sample and *L*_2_, *a*_2_, and *b*_2_ are color parameters for the BM-based fermented drink with different BP content, respectively, after a specified period of time (weeks); (Equation (1)).

(1) $$\Delta E=\sqrt{({L_{1}-L_{2})}^{2}+({a_{1}-a_{2})}^{2}+({b_{1}-b_{2})}^{2}}$$

If ΔE < 1, the difference between colors is not visible to the human eye. When 1 < ΔE < 3, the color differences are not considered significant to the human eye. If ΔE > 3, the color differences are considered visible to the human eye [21].

#### 2.2.4. UV-Vis Spectroscopy

UV-Vis experiments were conducted using a UV-1800 UV-VIS spectrophotometer (Shimadzu, Kyoto, Japan). To determine the total polyphenols (TPC) and antioxidant activity of BM-based fermented samples with different BP content, extraction was performed by mixing 10 g of the sample with 30 mL of an 80:20 methanol:water solution on a magnetic stirrer for 30 min. The mixture was then transferred to a centrifuge tube and centrifuged at 8000 rpm at 4 °C for 30 min [9].

##### Determination of Total Polyphenols

A volume of 0.3 mL of supernatant was transferred into a tube and mixed with 5 mL of Folin-Ciocalteu reagent (diluted 1:10). After 5 min, 1.5 mL of 6% Na_2_CO_3_ was added. The solutions were mixed with a vortex and left in the dark for 90 min. After the reaction time, the absorbance of the samples at 760 nm was measured using a UV-1800 UV-VIS spectrophotometer (Shimadzu, Japan). Results were expressed as µg gallic acid equivalent (GAE) per ml of fresh sample weight [9].

##### Determination of Antioxidant Activity

The free radical scavenging activity of BM-fortified samples was assessed following the method outlined by Barkallah et al. [22], employing 1,1-diphenyl-2-picrylhydrazyl (DPPH) as the indicator. Absorbance was measured at 517 nm using the same spectrophotometer. The results of all antioxidant activity determinations were expressed in mmol Trolox equivalents (TE), as Trolox is a stable antioxidant widely used as a standard for measuring antioxidative activity. A calibration curve ranging from 0.01 to 5.00 mmol of Trolox was used for the quantification of these activities.

#### 2.2.5. Microbiological Analysis

To begin microbial count analysis, 10 g samples of each buttermilk-based fermented drink were refrigerated and then homogenized in 90 mL of a diluent solution comprising 0.85% sodium chloride and 0.1% tryptone. Following homogenization, serial 10-fold dilutions were meticulously prepared for further examination. Enumeration of *Enterobacteria* was performed on Violet Red Bile Glucose (VRBG) Agar (Condalab, Madrid, Spain, CAT 1092.00) according to ISO standard [23]. Yeast and mold analyses were conducted in accordance with ISO protocols [24].

#### 2.2.6. Sensory Evaluation

The sensory evaluation of the BM-based fermented samples involved 20 semi-trained assessors who utilized a hedonic scale analysis. Approximately 10 g of homogenized samples were placed into glass cups labeled with randomized three-digit numbers and served chilled at 4 °C. Assessors rated the BM-based fermented drinks on a scale from 1 to 9, where 1 indicated “extremely dislike” and 9 indicated “extremely like”, considering attributes such as flavor, mouth-feel, appearance, texture, and overall acceptance. The sensory analysis was conducted solely on the first day following the BM-based fermented drinks production.

#### 2.2.7. Statistical Analysis

The results are presented as mean ± standard deviation. For physicochemical and microbiological parameters, *n* = 3 replicates were used, while, for the sensory evaluation, *n* = 20 assessors participated. Statistical analysis was conducted using factorial analysis of variance (2-way ANOVA), followed by Fisher’s least significant difference (LSD) test, to assess the main effects (BP addition and storage) as well as their interaction effects on all the analyzed traits. XLSTAT software version 2019.2.2 (Addinsoft, New York, NY, USA) was employed for statistical analysis. The significance level was set at *p* ≤ 0.05.

## 3. Results and Discussion

### 3.1. Chemical Characteristics

**Figure 2:**
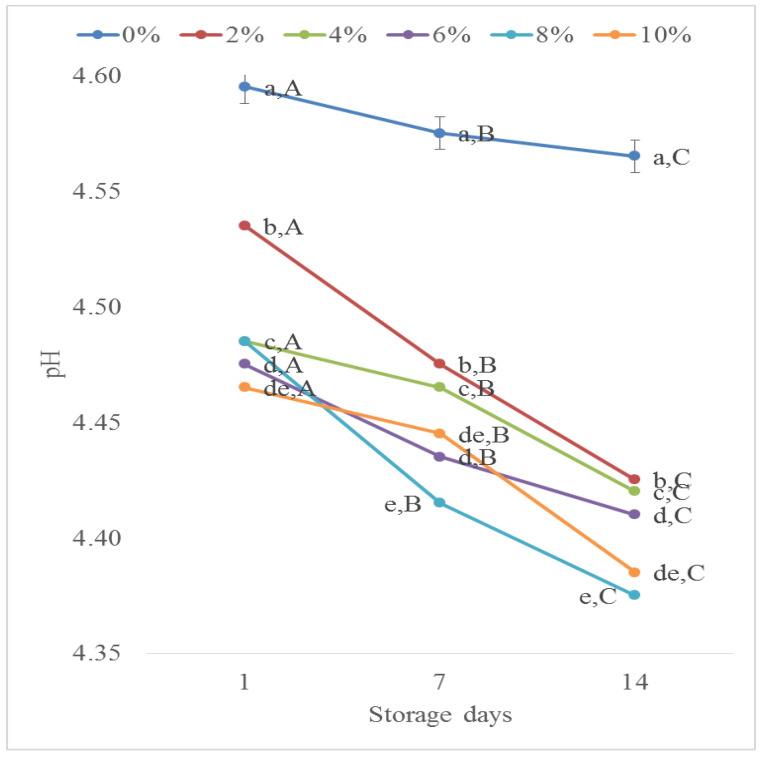
pH variation during storage of buttermilk fortified with different percentages of blueberry pomace. Note: Small letters refer to statistically significant differences (*p* < 0.05) between the samples with different quantities of BP; Capital letters refer to statistically significant differences (*p* < 0.05) between storage days.

During production, pH value is a critical step in determining the safety and shelf life of a dairy product. On the first day, the pH of the plain BM (0%—without blueberry pomace) was 4.60. The addition of BP significantly decreased the pH value of the BM-based fermented samples (*p* < 0.05); namely, the BM with 10% BP had a pH of 4.47 on the first day of storage. Nevertheless, the pH value of both the plain and the fortified BM with BP decreased with the storage time (*p* < 0.05). Eventually, on Day 14 of the examination, for the plain BM, the pH was 4.57 and for the blueberry pomace-fortified BM, it was 4.39 (10% BP fortified BM) (Figure 2). This trend mirrors findings in other food applications, such as a blueberry flower fermented drink [25] and a berry fruit pomace-fortified mustard [26], where a similar inverse correlation with the inclusion of blueberry flowers or pomace was observed. Similarly, the addition of Spirulina platensis to BM samples led to a notable reduction in the time required to reach a pH of 4.5 [27].

Dry matter and WHC exhibited a significant increase with the incorporation of BP, both during storage (*p* < 0.05) (Table 1). Initially, the dry matter content in our research ranged from 11.6% ± 0.02 to 19.6% ± 0.01 on the first day of post-production, with a subsequent increase attributed to the addition of BP. This elevation in dry matter content is likely influenced by the composition of the raw materials [28]. Similarly, WHC initially ranged from 24.6% ± 0.07 to 34.9% ± 0.02 and showed a continuous increase during storage across all samples. This augmentation can be associated with the rise in total solids content, which enhances WHC and reduces syneresis [29].

### 3.2. Color

Food selection is significantly influenced by its color and appearance, which serve as key indicators of food quality; often, color is the primary sensory attribute perceived by consumers, directly impacting their purchasing decisions [30]. Incorporating BP into the BM samples resulted in notable alterations in color parameters, including a decrease in the L* parameter value (*p* < 0.05). Furthermore, extended storage at 4 °C did not result in a significant decrease (*p* > 0.05) in the L* parameter values for the BP-fortified samples. Similar trends were observed with the yellowness coordinate (b*), whereby, compared to plain BM, the average b* value was 0.36 ± 7.27 on the first day after production, while, when fortified with 10% BP, the b* value increased to 9.69 ± 0.18. Additionally, both the blueberry concentration and storage duration significantly influenced the b* values, along with their interactions. Conversely, no statistically significant changes were detected in the redness coordinate (a*) for fortified BM samples, consistent with findings by Starkute et al. [31].

The color of fermented beverages often derives from pigments present in the raw materials used, as noted by Olukomaiya et al. [32]. Additionally, the lightness index of fermented milk products is affected by acidity, with a decrease in pH correlating with a decrease in lightness [33,34]. These findings align with our own results, which demonstrate that fortifying BM with BP leads to reduced acidity and a decrease in the L* parameter (Table 2), with more significant changes observed at 10% fortification with blueberry pomace. Furthermore, it is important to note that color parameters can vary over time, and storage duration can also influence these parameters [35]. Moreover, the size of fat globules and protein particles can significantly influence the brightness level [36].

The total color change (ΔE) between the control BM fermented drink sample and the BM fermented drink samples with varying BP content was also determined. According to the results presented in Table 2, it can be observed that these parameters changed during the storage period of the samples and with the fortification with BP. In both cases, the color differences are greater than 3, which are considered visible to the human eye [21].

### 3.3. Total Polyphenols and Antioxidant Activity of Buttermilk with Different Amounts of Blueberry Pomace

**Figure 3:**
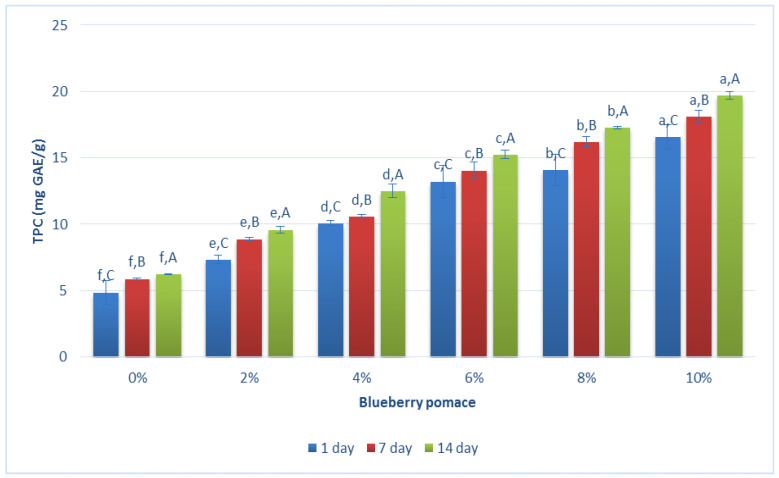
Total phenolic content (expressed as mg GAE/g) during storage days of buttermilk with different amounts of blueberry pomace. Small letters refer to statistically significant differences (*p* < 0.05) between the samples with different quantities of BP; Capital letters refer to statistically significant differences (*p* < 0.05) between storage days.

TPC without BP ranged from 4.9 ± 0.9 to 6.2 ± 0.04 mg GAE/g during the storage period (Figure 3), attributed to naturally occurring phenolic compounds in cultured BM-based fermented drinks. Similarly, Saberi et al. [37] observed significantly higher total phenolic content values in fortified yogurts with grape pomace and flaxseed oil compared to a control yogurt during storage. Similar tendencies were observed in the study of Starkute et al. [31], where the total phenolic content and antioxidant activity significantly increased in enriched samples, demonstrating the potential of raspberry, blueberry, and elderberry industry by-products for enhancing the nutritional and functional properties of unripened cow milk curd cheese. Various factors such as berry variety, cultivation region, and extraction and drying methods can influence phenolic compound levels [38]. The literature highlights blueberries as not only rich in fiber, minerals, and vitamins, but also a significant source of antioxidants, known for their documented health-promoting effects [13,14]. The addition of blueberry pomace (10%) significantly (*p* < 0.05) enhanced the total phenolic content to 16.6 ± 0.9 mg GAE/g, as compared to the plain BM-based fermented drink (4.9 ± 0.9 mg GAE/g). Although TPC levels can increase with storage time within each sample, it is important to note that the Folin-Ciocalteau assay, commonly used to measure TPC, assesses the total reducing capacity of the sample, not just TPC [39]. Additionally, the temporary decrease in TPC observed in yogurt could be due to the decomposition of polymeric phenolics in the presence of lactic acid bacteria during refrigerated storage [40].

**Figure 4:**
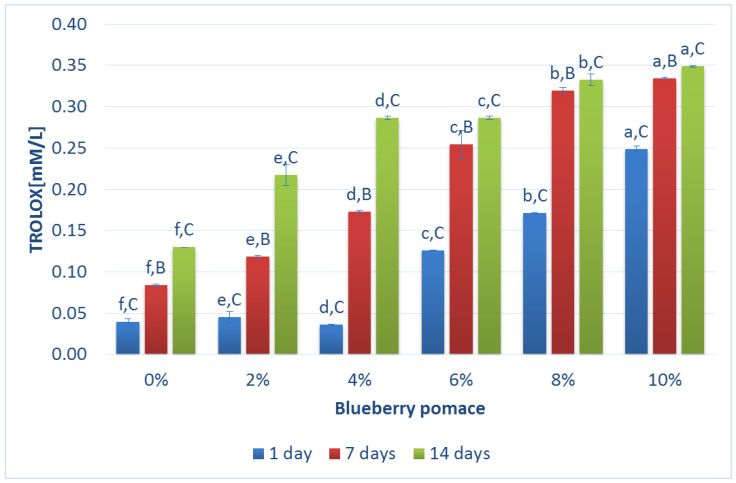
Antioxidant activity-AOA (expressed in Trolox mM/L) during storage days of buttermilk with different amounts of blueberry pomace. Small letters refer to statistically significant differences (*p* < 0.05) between the samples with different quantities of BP; Capital letters refer to statistically significant differences (*p* < 0.05) between storage days.

During the storage of both plain and fortified BM-based fermented samples, the antioxidant activity, expressed as the Trolox equivalent antioxidant capacity (TEAC), was estimated. With an increase in the amount of BP in the samples, the antioxidant activity also rose (from 0.04 ± 0.0% in plain BM-based fermented drinks to 0.25 ± 0.0% for 10% BP in BM-based fermented drinks) on the first day after production (Figure 4). Among six BM-based fermented samples, the radical scavenging activity (mmol of TA/l) was the highest in BM fortified with 10% BP. Plant polyphenols have been reported to effectively enhance the antioxidant activity of fermented dairy products [8,9]. It is worth mentioning that BP is rich in polyphenols with well-known antioxidant activity [41]. The greater the addition of BP, the higher the observed radical scavenging activity. This suggests that BP, containing numerous polyphenols, significantly boosts the antioxidant activity of BM. Consequently, BM containing BP exhibits substantial antioxidant activity, positioning it as a novel dairy product with notable health benefits. Our research agrees with those of previous reports, which described that the addition of berry industry by-products contained higher radical scavenging activity due to the higher amount of phenolic compounds [31,37]. Also, similar findings were presented by Najgebauer-Lejko and Sady [42] where BM flavored with strawberry and rhubarb exhibited higher radical scavenging activity (0.88 mmol of TE/kg) compared with the control sample. Additionally, antioxidant properties in fermented milk come from compounds such as casein, whey proteins, peptides, amino acids, coenzyme Q10, enzymatic systems, and lactic acid bacteria. In BM, lipophilic antioxidants are effective due to their variety, thermal stability, and synergistic interactions with hydrophilic antioxidants, protecting against undesirable compounds during processing [42]. On the other hand, Liu et al. [26] noticed that adding blueberry flower pulp to yogurt significantly increased the antioxidant properties of the yogurt. Throughout storage, these benefits remained relatively stable, with only minor fluctuations. However, according to their research, the addition of blueberry flower pulp led to a decrease in antioxidant activity. This could be attributed to the gradual decline of polyphenols, likely influenced by bacterial metabolic processes. Polyphenol oxidase might also play a role in this observed phenomenon [43].

### 3.4. Microbiological Analysis

The perishability of dairy products is primarily governed by the microbiological quality of the product [44]. Throughout the 14-day storage period at 4 °C, none of the samples exhibited any signs of mold, yeast, or *Enterobacteriaceae*, which are considered indicative of hygiene standards. This absence of bacteria suggests that the BM-based fermented samples remained secure and uncontaminated, reflecting the adherence to clean and hygienic processing conditions. Similar findings were reported by Rose et al. [27], who studied cultured buttermilk fortified with Spirulina platensis, and by Parekh et al. [44], who investigated cultured buttermilk prepared by incorporating paneer whey.

### 3.5. Sensory Analysis of Buttermilk with Different Amounts of Blueberry Pomace

**Figure 5:**
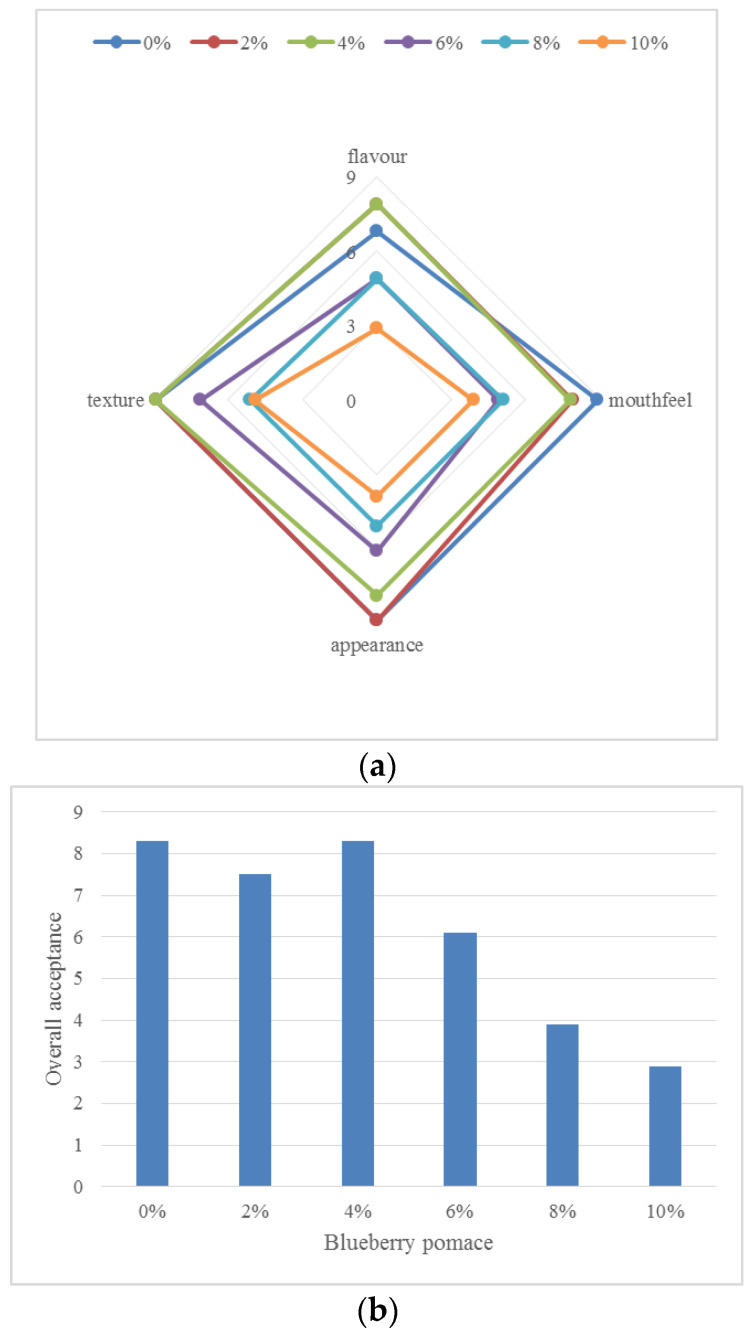
(**a**). Sensory evaluation of BM fortified with different concentrations of BP, assessing flavor, mouth-feel, appearance, and texture. (**b**). Overall acceptance of BM fortified with different concentrations of BP.

Various factors, such as the composition and quality of the product, influenced the sensory analysis [45]. The ANOVA (not presented) highlighted significant differences (*p* < 0.05) for all sensory parameters between the control sample (BM without BP) and other BMs with BP. (Figure 5a,b). The BM samples formulated with 2% and 4% BP received the highest sensory scores. Additionally, the highest overall acceptance score, averaging 8.3, was observed for the plain BM-based fermented drink and the 4% fortified BM-based fermented drink (Figure 5b). Significant differences were noted in the visual appearance, texture, and overall acceptance of BM-based fermented samples formulated with BP (6%, 8%, and 10%). A similar trend was observed in a previous study comparing Petit Suisse cheese made with and without blueberry bagasse powder [46]. While previous reports have highlighted the addition of BP to foods [47], there is limited research on its addition to dairy products. Liu and Lv [26] conducted a sensory analysis of yogurt containing blueberry flower pulp, finding that panelists preferred samples with 2–5 g of blueberry flower pulp over the control. These results align with our findings. Thus, incorporating blueberry-based ingredients improved the overall acceptability and sensory properties of the BM-based fermented drinks. Other findings suggest a decrease in sensory scores during storage, possibly due to the relationship between bacterial fermentation and the resulting acidity from oxidative decomposition of product ingredients [48].

## 4. Conclusions

This study demonstrates the potential of incorporating blueberry pomace into BM to valorize fruit by-products and create functional dairy products with enhanced nutritional and sensory properties. Significant improvements were observed in the physicochemical parameters, including pH, dry matter content, water holding capacity, and color. The addition of blueberry pomace significantly increased the total phenolic content and antioxidant activity, highlighting its health-promoting benefits. Microbiological analysis confirmed the safety of all samples, with no presence of *Enterobacteriaceae*, yeast, or molds. Sensory evaluation revealed that BM fortified with 2% and 4% blueberry pomace received the highest scores, indicating favorable consumer acceptance. However, samples with 6%, 8%, and 10% pomace had lower sensory quality. These findings suggest that incorporating blueberry pomace can enhance the nutritional profile, functional properties, and overall appeal of BM, contributing to sustainable practices in the dairy industry. Further research is needed to optimize pomace incorporation, assess long-term stability, and evaluate broader consumer preferences.

## Figures and Tables

**Table 1:** Mean values and standard deviation of dry matter—DM (%) and water holding capacity—WCH (%) on different percentages of BP in BM during storage days.

Parameters	BP (%)	Storage Period (Days)		
		1	7	14
**Dry matter (DM %)**	**0**	11.6 ± 0.02 ^f,C^	12.4 ± 0.04 ^f,B^	14.0 ± 0.02 ^f,A^
	**2**	13.0 ± 0.04 ^e,C^	14.8 ± 0.01 ^e,B^	16.2 ± 0.02 ^e,A^
	**4**	14.8 ± 0.04 ^d,C^	15.3 ± 0.01 ^d,B^	17.9 ± 0.04 ^d,A^
	**6**	16.2 ± 0.06 ^c,C^	17.4 ± 0.05 ^c,B^	20.1 ± 0.04 ^c,A^
	**8**	17.8 ± 0.06 ^b,C^	19.6 ± 0.07 ^b,B^	21.7 ± 0.07 ^b,A^
	**10**	19.6 ± 0.01 ^a,C^	20.1 ± 0.03 ^a,B^	23.7 ± 0.05 ^a,A^
**WHC (%)**	**0**	24.6 ± 0.07 ^f,C^	27.3 ± 0.02 ^f,B^	35.2 ± 0.04 ^f,A^
	**2**	26.0 ± 0.11 ^e,C^	28.9 ± 0.02 ^e,B^	35.6 ± 0.07 ^e,A^
	**4**	29.0 ± 0.04 ^d,C^	31.2 ± 0.05 ^d,B^	38.7 ± 0.02 ^d,A^
	**6**	31.8 ± 0.01 ^c,C^	34.2 ± 0.02 ^c,B^	41.8 ± 0.18 ^c,A^
	**8**	33.3 ± 0.12 ^b,C^	37.4 ± 0.02 ^b,B^	43.9 ± 0.04 ^b,A^
	**10**	34.9 ± 0.02 ^a,C^	36.7 ± 0.04 ^a,B^	45.4 ± 0.04 ^a,A^

Note: Small letters refer to statistically significant differences (*p* < 0.05) between the samples with different quantities of BP; Capital letters refer to statistically significant differences (*p* < 0.05) between storage days.

**Table 2:** Mean values and standard deviation of color coordinates (L*, a*, and b*) on different percentages of blueberry pomace in buttermilk during storage days.

Parameters	BP (%)	Storage Period (Days)		
		1	7	14
L*	**0**	73.9 ± 7.60 ^a,A^	79.9 ± 1.68 ^a,A^	78.7 ± 2.83 ^a,A^
	**2**	66.5 ± 0.60 ^b,A^	66.9 ± 0.37 ^b,A^	65.8 ± 0.35 ^b,A^
	**4**	61.0 ± 0.36 ^c,A^	60.2 ± 0.83 ^c,A^	58.9 ± 0.00 ^c,A^
	**6**	56.2 ± 0.08 ^d,A^	55.1 ± 0.13 ^d,A^	54.1 ± 0.30 ^d,A^
	**8**	51.1 ± 0.03 ^e,A^	49.9 ± 0.31 ^e,A^	50.4 ± 0.17 ^e,A^
	**10**	46.5 ± 0.34 ^f,A^	47.0 ± 0.17 ^f,A^	47.3 ± 0.55 ^f,A^
a*	**0**	3.32 ± 7.87 ^b,A^	−0.68 ± 3.56 ^b,A^	−15.13 ± 15.29 ^b,A^
	**2**	2.01 ± 0.08 ^a,A^	2.31 ± 0.05 ^a,A^	2.43 ± 0.11 ^a,A^
	**4**	3.46 ± 0.11 ^a,A^	3.45 ± 0.03 ^a,A^	3.60 ± 0.05 ^a,A^
	**6**	4.25 ± 0.02 ^a,A^	4.73 ± 0.07 ^a,A^	4.29 ± 0.18 ^a,A^
	**8**	5.40 ± 0.06 ^a,A^	5.83 ± 0.09 ^a,A^	5.18 ± 0.13 ^a,A^
	**10**	6.08 ± 0.04 ^a,A^	6.22 ± 0.17 ^a,A^	5.82 ± 0.11 ^a,A^
b*	**0**	0.36 ± 7.27 ^d,A^	−2.98 ± 2.68 ^d,A^	−0.44 ± 3.25 ^d,A^
	**2**	2.96 ± 0.05 ^c,A^	2.44 ± 0.25 ^c,A^	4.04 ± 0.21 ^c,A^
	**4**	5.54 ± 0.53 ^b,c,A^	5.83 ± 0.09 ^b,c,A^	5.65 ± 0.08 ^b,c,A^
	**6**	7.34 ± 0.03 ^a,b,A^	7.95 ± 0.12 ^a,b,A^	7.43 ± 0.44 ^a,b,A^
	**8**	8.62 ± 0.07 ^a,,A^	9.63 ± 0.20 ^a,A^	8.36 ± 0.16 ^a,A^
	**10**	9.69 ± 0.18 ^a,A^	10.09 ± 0.03 ^a,A^	9.11 ± 0.25 ^a,A^
**ΔE**	**2**	11.4	14.7	23.8
	**4**	15.6	22.2	29.5
	**6**	25.2	27.9	33.8
	**8**	25.2	33.1	37.3
	**10**	29.3	36.12	40.3

Note: Small letters refer to statistically significant differences (*p* < 0.05) between the samples with different quantities of BP; Capital letters refer to statistically significant differences (*p* < 0.05) between storage days. ΔE—difference between control samples and samples with different quantities of BP.

## Data Availability

The original contributions presented in the study are included in the article, further inquiries can be directed to the corresponding author.
